# Endoscopic Stenting and Sphincterotomy of the Minor Papilla in Symptomatic Pancreas Divisum: Results and Complications

**DOI:** 10.1155/DTE.1.131

**Published:** 1995

**Authors:** Seth A. Cohen, Frederick D. Rutkovsky, Jerome H. Siegel, Franklin E. Kasmin

**Affiliations:** 1 Section of Endoscopy Beth Israel Medical Center North Division New York New York USA; 2 60 East End Avenue New York NY 10028 USA

## Abstract

Pancreas divisum has been postulated as a cause of acute pancreatitis and a chronic pain syndrome in a small subgroup of patients and can be treated with endoscopic dorsal pancreatic duct stent placement and minor papilla sphincterotomy. Twenty patients (9 with at least one attack of idiopathic pancreatitis, and 11 with severe pancreatic-type pain) were treated endoscopically. Dorsal duct stents were placed in 19 patients with subsequent needle knife sphincterotomy of the minor papilla over the stent. Clinical response was judged by comparison of symptoms (using a 0-to-l0 scale and the patient's overall assessment). The symptom score improved from 9.3 to 5.1 in the pancreatitis group and from 9.3 to 5.7 in the pain group. A good clinical response was observed in 3 of 7 patients in the pancreatitis group and in 6 of 11 in the pain group at a mean follow-up of 22 months. Complications of sphincterotomy were limited to pancreatitis in 6 patients (29%), 5 mild and 1 moderate according to published criteria. No patient required more than 4 days hospitalization. Two of 39 stents migrated into the pancreas, and another stent fractured and remained lodged in the pancreas. Eight of 9 patients evaluated demonstrated new morphologic duct changes on follow-up pancreatograms. Endoscopic stenting and sphincterotomy of the minor papilla are feasible and may be effective in some patients with pancreas divisum but carries a significant complication rate. The subjective improvement in patients with chronic pain warrants further controlled study.


*Diagnostic and Therapeutic Endoscopy*, 1995, Vol. 1, pp. 131–139
Reprints available directly from the publisher
Photocopying permitted by license only

© 1995 Harwood Academic Publishers GmbH
Printed in Malaysia

### Endoscopic Stenting and Sphincterotomy of the Minor Papilla in Symptomatic Pancreas Divisum: Results and Complications

SETH A. COHEN, FREDERICK D. RUTKOVSKY, JEROME H. SIEGEL, and FRANKLIN E. KASMIN

*Section of Endoscopy, Beth Israel Medical Center, North Division, New York, New York*

*(Received January 27, 1994; in final form, April 4, 1994)*

Pancreas divisum has been postulated as a cause of acute pancreatitis and a chronic pain syndrome in a small subgroup of patients and can be treated with endoscopic dorsal pancreatic duct stent placement and minor papilla sphincterotomy. Twenty patients (9 with at least one attack of idiopathic pancreatitis, and 11 with severe pancreatic-type pain) were treated endoscopically. Dorsal duct stents were placed in 19 patients with subsequent needle knife sphincterotomy of the minor papilla over the stent. Clinical response was judged by comparison of symptoms (using a 0-to-10 scale and the patient's overall assessment). The symptom score improved from 9.3 to 5.1 in the pancreatitis group and from 9.3 to 5.7 in the pain group. A good clinical response was observed in 3 of 7 patients in the pancreatitis group and in 6 of 11 in the pain group at a mean follow-up of 22 months. Complications of sphincterotomy were limited to pancreatitis in 6 patients (29%), 5 mild and 1 moderate according to published criteria. No patient required more than 4 days hospitalization. Two of 39 stents migrated into the pancreas, and another stent fractured and remained lodged in the pancreas. Eight of 9 patients evaluated demonstrated new morphologic duct changes on follow-up pancreatograms. Endoscopic stenting and sphincterotomy of the minor papilla are feasible and may be effective in some patients with pancreas divisum but carries a significant complication rate. The subjective improvement in patients with chronic pain warrants further controlled study.

**KEY WORDS:** pancreas divisum, recurrent pancreatitis, endoscopic stenting, pancreatic sphincterotomy

### INTRODUCTION

Pancreas divisum is the most common congenital malformation of the pancreas, arising when the embryonic dorsal and ventral anlagen fail to fuse in the usual manner. As a result, most of the head and all of the body and tail of the pancreas drain via the dorsal duct and through the minor papilla. It is estimated from autopsy series that pancreas divisum is present in up to 9% of the population (1).

Pancreas divisum has been proposed as a cause for acute pancreatitis and a pancreatic pain syndrome (2–3). This hypothesis has been both supported and contradicted during the past 10 years (4–7). The pathogenesis is assumed to be a relative obstruction of pancreatic flow through the small minor papilla.

Address for correspondence: Jerome H. Siegel, MD, 60 East End Avenue, New York, NY 10028.

A variety of surgical and endoscopic interventions have been performed to relieve the proposed ductal obstruction and improve pancreatic drainage with variable to good results. (8–14). We report the technical feasibility, response to therapy, and complications in a retrospective study in which a combination of endoscopic stenting and sphincterotomy of the minor papilla was performed in 20 patients with symptomatic pancreas divisum.

### PATIENTS AND METHODS

Our records from 1987 to 1991 were searched to identify all patients with symptomatic pancreas divisum and had undergone sphincterotomy of the minor papilla. Twenty-one patients were identified, but one patient who was ultimately found to have carcinoma was excluded. The remaining 20 patients (7 men and 13 women, mean age, 43.3 years) with symptomatic pancreas divisum who were treated with pancreatic stenting and endoscopic sphincterotomy of the minor papilla make up the basis of this report. Four of these patients were included in an earlier report (14). The diagnosis of pancreas divisum was established by pancreatography in all patients. No patient had been previously treated endoscopically or surgically.

Eight patients reported at least two documented attacks of pancreatitis, whereas 1 patient had one attack (4 women and 5 men, mean age, 50.7 years). Pancreatitis was defined as severe abdominal pain associated with an elevation of serum amylase to more than three times the normal level or evidence of pancreatitis on computed tomography (CT). The majority of these patients had frequent episodes of epigastric pain that were similar in character to, but were not, attacks of acute pancreatitis. Known causes of pancreatitis were excluded including pancreatitis caused by gallstones, alcohol consumption, hypertriglyceridemia, hypercalcemia, medications, and heredity. Six patients had had their gallbladders removed; the remaining 3 patients did not have cholelithiasis by sonography and cholangiography.

Eleven patients (9 women and 2 men, mean age, 36.5 years) had disabling pancreatic-type pain without pancreatitis ("pain-only" group). Serum amylase values were normal or less than twice normal. The pancreas was normal by sonography or CT. All patients were thoroughly evaluated to exclude other causes of abdominal pain such as cholelithiasis, choledocholithiasis, and peptic ulcer disease. Four patients had intact gallbladders. Sphincter of Oddi manometry was performed in 2 patients, and pressures were within the normal range.

The general outline of endoscopic treatment was as follows: endoscopic retrograde cholangiopancreatography (ERCP) and stent placement at time zero; sphincterotomy and stent exchange at 2 to 3 months; stent removal at 4 to 6 months.

Each patient underwent ERCP with visualization of the biliary tree and usually the ventral pancreas. The dorsal pancreatic duct was opacified (Fig. 1) in every patient using a needle-tipped catheter (Wilson-Cook), to cannulate the minor papilla. No patient had dorsal changes suggestive of chronic pancreatitis. A 0.035-inch guidewire was inserted into the dorsal pancreatic duct via the minor papilla (Figs 2 and 3), and when possible the orifice was dilated with a tapered 4 to 7 French dilation catheter (Wilson-Cook); 0.018 – and 0.025-inch guidewires were not employed. It was possible to place 5 or 7 French pancreatic stents in 17 of 20 patients; either strait "Geenen" or C-loop "Siegel" stents, both with multiple side holes were used (Wilson-Cook) (Figs 3 and 4). In 3 patients this was not possible, and a *de novo* precut sphincterotomy was performed using a needle-knife (Wilson-Cook) (Fig. 5). One of these patients improved and required no further intervention; the other 2 returned for successful stent placement and extension of the sphincterotomy.

Eighteen patients returned after stent insertion and underwent sphincterotomy of the minor papilla with the stent in place (mean, 5.1 months; range, 1 to 22 months). A needle-knife sphincterotome was used to cut 3 to 5 mm upward and 2 to 3 mm deep in the 11 o'clock direction exposing the pancreatic prosthesis (Fig. 6). The initial stent then was removed and replaced with a new stent to prevent restenosis of the orifice during healing. Removed stents were almost all occluded. Placing a new stent provides pancreatic drainage despite the presence of inflammation and edema that occur following sphincterotomy. Pancreatic stents were removed, usually on a outpatient basis, a mean of 3.6 months after insertion.

Hospital and office records were reviewed to determine complications and hospital stay for all patients after sphincterotomy of the minor papilla. Postprocedure complications were defined according to the recent consensus conference criteria (15). Patients were seen in follow-up in the office or by the referring gastroenterologist under our direction.

Follow-up for 18 of 20 patients was obtained by a single interviewer not involved with the endoscopic procedures or patient care. Patients were asked to classify pain before and after therapy on a linear scale of 0 (none) to 10 (most severe). Patients also were asked to rate their response to therapy by choosing from 5 adjectives: worse, same, somewhat better, much better, or completely better. "Somewhat better" was not considered a positive response.

Radiographs obtained before and at the time of pancreatic stent removal were available for 9 patients and were reviewed blindly by a radiologist for possible ductal changes. No patient had follow-up pancreatograms after stent removal.

A Pearson $\chi^2$ test was used to compare the proportion of patient outcome between groups. A paired $t$ test was used to compare the mean symptom scores of patients within each group. The MANOVA test was used to analyze the variance of pre- and postprocedure symptom scores of patients by group.

### RESULTS

Follow-up was available for 18 patients (7 in the pancreatitis group, and 11 in the pain-only group) at a mean of 22 months after initial stent placement (range, 4 to 57 months). Five (71%) of 7 patients in the pancreatitis group clinically responded to initial stent placement, but after a

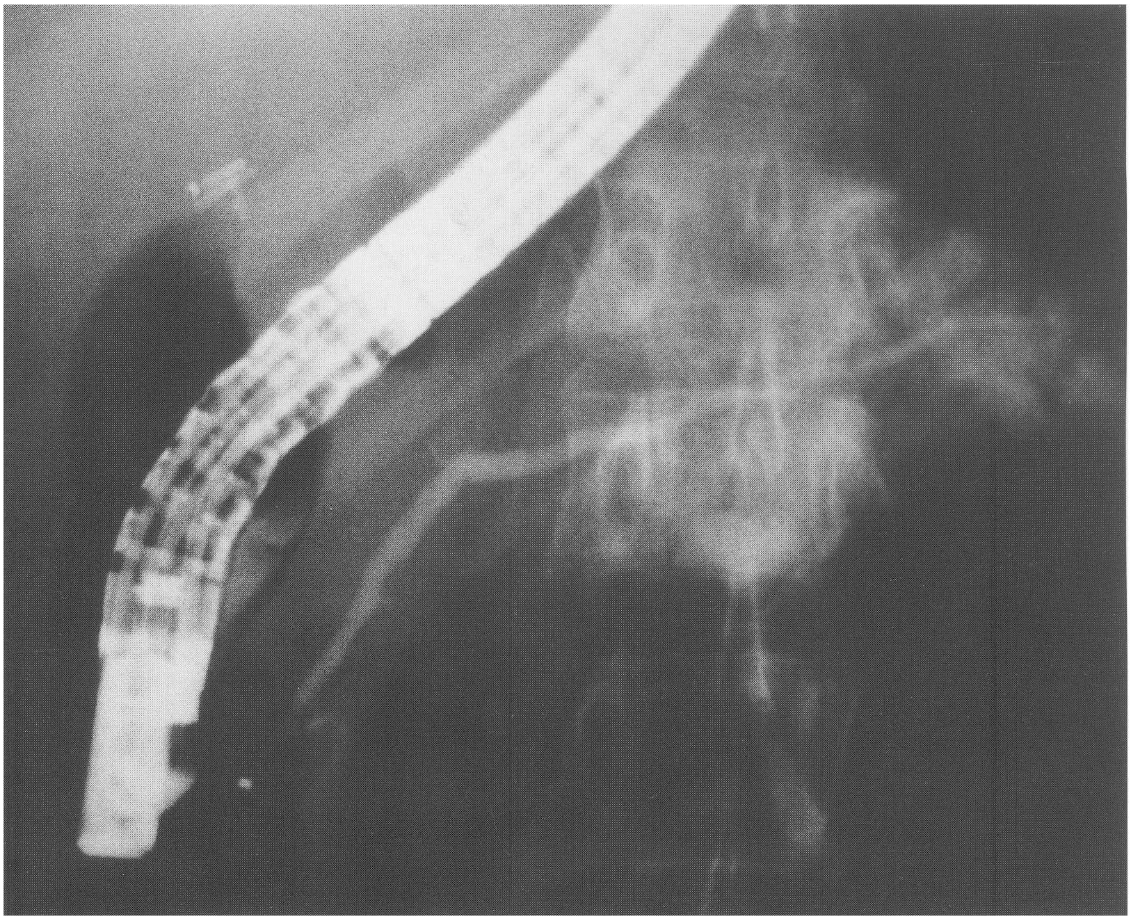

**Figure 1** Endoscopic retrograde pancreatogram demonstrating a dorsal pancreatic duct in a patient with pancreas divisum. Overfilling of the tail is present.

mean of 13.7 months only 3 (43%) of 7 were "much better" or "completely better". Eight (73%) of 11 patients in the pain-only group gained relief with the initial stent, but at mean follow-up of 25.4 months this decreased to 6 (55%) of 11 patients (Table 1). One of the 7 patients in the pancreatitis group experienced an attack of pancreatitis. The mean symptom score after treatment was significantly reduced in both groups: 9.0 ± 0.6 (± standard error) to 5.0 ± 1.3 ($p < 0.018$) in the pancreatitis group, and 9.2 ± 0.3 to 4.5 ± 1.1 ($p < 0.001$) in the pain-only group. There was, however, no statistical difference in the clinical improvement between these two groups.

**Table 1** Symptomatic response to dorsal duct stenting and minor papilla sphincterotomy in patients with pancreas divisum

| | *Pancreatitis group (N = 7)* | *Pain-only group (N = II)* |
|---|---|---|
| better | 2 | 1 |
| Much better | 1 | 5 |
| Somewhat better | 4 | 4 |
| Same | 0 | 1 |
| Worse | 0 | 0 |

Six (29%) of 21 patients undergoing sphincterotomy experienced pancreatitis: 5 mild, 1 moderate according to published criteria (15). None of the 3 patients in the precut group experienced complications. However, the pancreatitis was mild, and no patient was discharged later than the fourth day. The mean hospital stay for all patients was 2.1 days (range, 1 to 4). There were no severe complications of pancreatitis, i.e., phlegmon, abscess, pseudocyst, or sepsis. Likewise, no other complications such as perforation or hemorrhage occurred, and there was no mortality in this series.

A total of 39 stents were placed in 19 patients: 29 7 French stents varying in length from 5 to 7 cm, and 10 5 French stents usually 5 cm in length. Sixteen patients had 2 stents inserted during treatment, 2 patients had 3 stents, and 1 patient had a single stent. Stents migrated proximally into the pancreas in 2 patients (5.1%) of 39 stents. One stent was retrieved endoscopically, the other surgically. One stent fractured during retrieval, with part of the stent remaining lodged in the dorsal duct. The patient continues to have attacks of pancreatitis as before therapy and

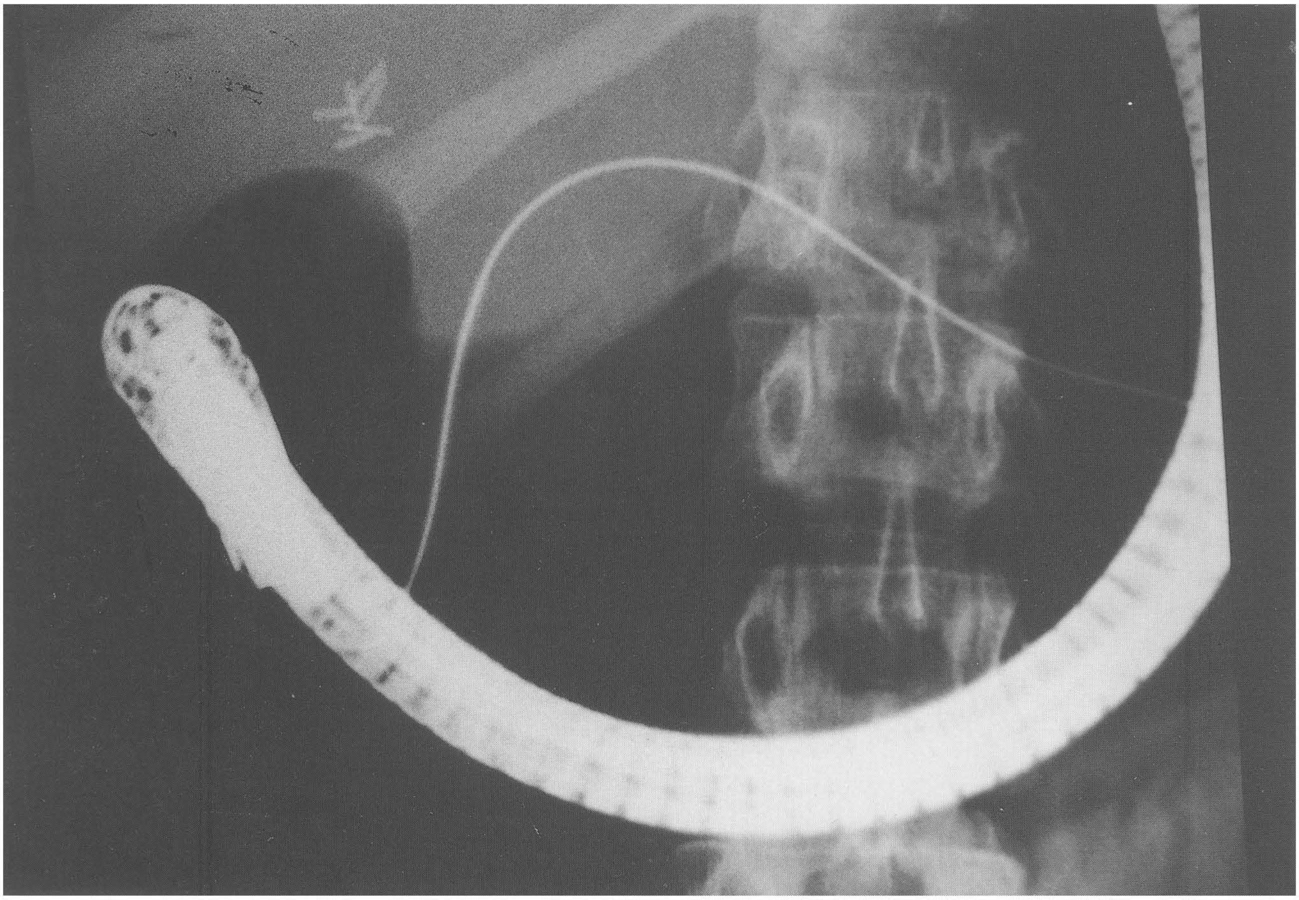

**Figure 2** A radiograph demonstrating cannulation of the dorsal pancreatic duct with a 0.035-in guidewire and tapered 7 French dilating catheter in the same patient.

refuses to undergo further intervention either endoscopic or surgical.

Radiographs were available for comparison of ductal changes in 9 patients prior to and at the time of stent removal. Stents remained in place from 2 to 11 months. All patients had normal pancreatograms before stenting. Eight of these 9 patients demonstrated morphologic duct changes on follow-up pancreatograms. All 8 patients had increases in the diameter of the dorsal duct of at least 75%, 2 patients demonstrated new strictures of the main pancreatic duct, and 7 patients had secondary branch changes.

### DISCUSSION

The pathogenesis of acute pancreatitis and a pancreatic pain syndrome in patients with pancreas divisum is not understood completely. Multiple surgical and endoscopic drainage procedures have been performed to treat these conditions based on the hypothesis that the minor papilla is too small to accommodate the volume of pancreatic secretion. In an early report, endoscopic sphincterotomy of the minor papilla proved to be technically difficult, with a successful outcome in only 5 of 12 patients, and ineffective, with a good outcome in only 1 patient (16). The data for surgical treatment, drawn from a small number of series reported by a few surgeons, show a good outcome in 74% to 93% of patients with pancreatitis after sphincteroplasty or sphincterotomy of the minor papilla. Fewer patients, approximately 38%, with the pancreatic pain syndrome derive benefit from surgery (9–10). On the other hand, patients with pancreas divisum and chronic pancreatitis can be effectively treated with resection like other patients with chronic pancreatitis (8).

Because of advances in technique and promising results of preliminary reports, endoscopic therapy for symptomatic pancreas divisum has generated much interest (17–18). Initially, in our earlier reports, we performed endoscopic dilation and stent placement without sphincterotomy in these patients and referred those who had recurrent or persistent symptoms after endoscopic therapy to (11, 14) surgery. Relief of symptoms after stent

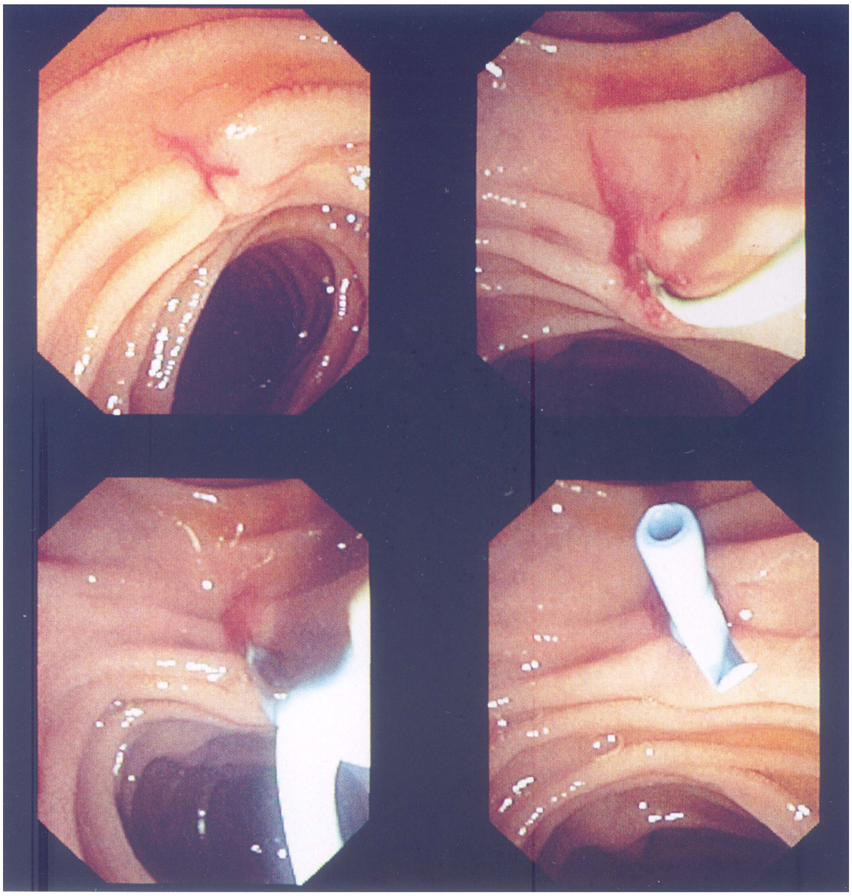

**Figure 3** Videoendoscopic sequence showing stent placement in the dorsal pancreatic duct via the minor papilla. (*Upper left*) the minor papilla, (*upper right*) cannulation with a 0.035-in guidewire, (*lower panels*) a straight 7 French stent as it is pushed into the dorsal duct, and the guidewire is removed.

placement into the dorsal duct predicted better clinical outcome in those patients who eventually underwent surgery. To achieve definitive therapy without surgery, we began performing endoscopic sphincterotomy of the minor papilla. The current report summarizes our recent experience.

The classification of patients with symptomatic pancreas divisum into those with documented pancreatitis and those with pain only has been reported to be important in predicting the clinical response to both surgical or endoscopic drainage (9–10, 13). No pathophysiologic explanation has been offered for the different responses to therapy between these two groups except the obvious fact that patients with pancreatitis exhibit definite evidence of pancreatic disease, whereas in patients with pain only, the evidence is circumstantial.

We observed clinical improvement with the initial stent in 71% of the pancreatitis patients and 73% of the pain-only patients. This decreased with follow-up to 43% and 55%, respectively. Initial clinical response to stenting was a favorable prognostic sign but did not assure long-term relief from endoscopic therapy. Comparing our response rates with those of other published studies is fraught with difficulty because of different methodology and the subjective nature of symptoms. Lans and colleagues reported a 50% or greater symptomatic improvement in 9 (90%) of 10 patients with pancreatitis after dorsal duct stenting, as opposed to only 1 (11%) of 9 in the control group (12). Lehman *et al.*, reported clinical benefit in 13 (76.5%) of 17 patients with pancreatitis treated with stents and sphincterotomy (13), whereas Barkun and associates related marked improvement in 9 (45%) of 20 patients treated with dilation and stenting (19). More recently, Coleman reported that stenting with or without sphincterotomy was effective in 10 (77%) of 13 of patients with acute recurrent pancreatitis.

The significant reduction of symptoms in 55% of our patients with a pancreatic pain syndrome is better than the 27% reported by Lehman *et al.* (13), or the 20% efficacy reported by Coleman for endoscopic treatment (20), and similar to the best surgical results of 56% reported by Warshaw (9). This finding is limited, however, by lack of

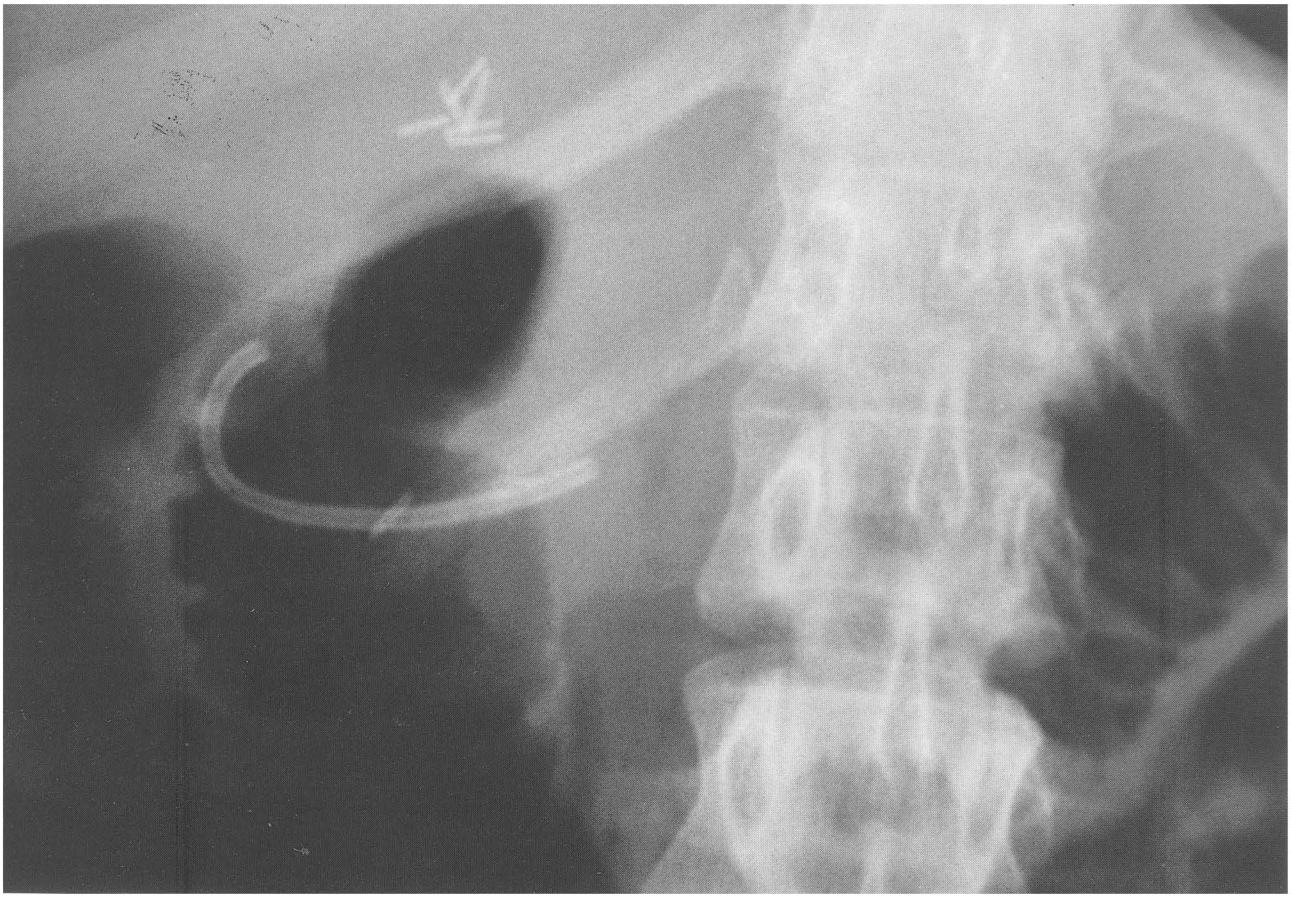

**Figure 4** A radiograph of a C-loop stent in the dorsal pancreatic duct after insertion (the same patient as in Figure 1 and 2).

a control group to determine the placebo effect of sham endoscopic therapy.

Our results have to be evaluated carefully because the patients were polled retrospectively concerning their symptoms, which has methodologic weaknesses. Optimally, objective parameters such as narcotic use and hospitalizations should be measured. Despite these shortcomings, we believe these results are promising and that pancreas divisum patients with pain deserve careful study. To date, only one series has compared minor papilla stenting and sphincterotomy versus no therapy in a controlled fashion in this population of patients (21). It showed that 44% of patients treated endoscopically clinically improved compared with only 24% of control patients and a trend towards a reduction of hospital days required for symptoms.

We were not able to demonstrate a difference in outcome between patients classified as pancreatitis and pain only. This lack of difference is bothersome and may be related to the small number of patients.

The incidence of pancreatitis after endoscopic sphincterotomy was 29%, with 5 mild and 1 moderate. The mean hospital stay in this study was 2.1 days. There were no serious complications such as phlegmon, necrosis, pseudocyst, or sepsis in our series. Direct manipulation of the pancreas at ERCP is expected to contribute to hyperamylasemia and possible pancreatitis, but as others have reported, this is usually not severe (22). These results are in keeping with our past experience of dorsal duct stenting in more than 90 patients with symptomatic pancreas divisum (11, 14). It must be emphasized that ERCP is an invasive procedure with a small but real incidence of severe, even fatal complications. One case of fatal post-ERCP pancreatitis has been reported in a patient with pancreas divisum after unsuccessful cannulation (13). This is an inherent risk of the procedure and not specifically related to treatment of pancreas divisum. Overall, endoscopic sphincterotomy of the minor papilla can be performed without prohibitive mortality.

Migration of stents into the dorsal pancreatic duct occurred with 5% of stents (2 of 39). This is similar to the incidence reported by Johanson, who found that 14 (5.2%) of 267 pancreatic stents migrated proximally. Eleven (79%) migrated stents were successfully retrieved endo-

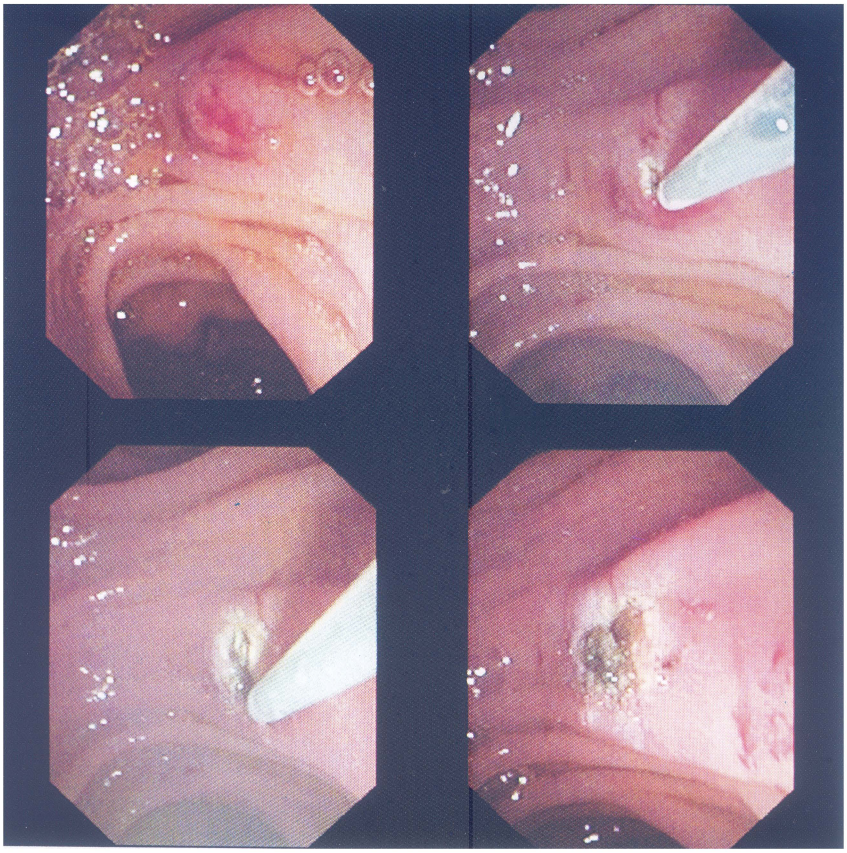

**Figure 5** Videoendoscopic sequence of a *de novo* needle knife sphincterotomy of the minor papilla. (*Upper left*) the minor papilla, (*upper right* and *lower left*) cutting with the needle knife sphincterotome, (*lower right*) after sphincterotomy.

scopically and 1 (7%) required surgical retrieval. Thus, migrated stents are usually retrievable with a basket or balloon but may require surgery for removal (24). One stent in the current series fractured during its retrieval. This is unusual; the stent may have been damaged during sphincterotomy and firmly adherent in the pancreas because of prolonged placement.

Stent migration was the most serious morbidity in this series: one patient underwent surgery because of persistent symptoms and a migrated stent, and the patient with the fractured stent still has a stent in place. To reduce stent migration, we have modified our pancreatic stents to include only a single proximal barb and a C-loop in the duodenum to prevent inward migration (Figure 4); Geenen and associates recommend removing the two proximal barbs from straight pancreatic stents and have reported that this reduces the incidence of inward migration.

We observed morphologic ductal changes including ectasia and dilation simulating chronic pancreatitis in 89% of patients with available radiographs. These changes have been previously reported (24, 27–28) and are probably related to occlusion of the stent, obstruction of side branches caused by insufficient number of side holes, and trauma to the ductal epithelium. This high rate of ductal changes probably relates to the prolonged stent duration and to the fact that most stents were occluded at the time of removal, although only half of the patients were symptomatic. Stent associated pancreatic ductal changes are largely reversible and have not been shown to be of clinical significance (28). We speculate that many of these changes would regress if the pancreatograms were repeated 4 to 6 months after stent removal. Ductal changes may be minimized by leaving the stents in for shorter periods of time (13) (i.e., less than eight weeks), varying the length of the stents during each exchange (12), and placing stents with more side holes to allow better drainage. Nevertheless, the incidence of ductal changes are high enough to argue that pancreatic stenting be prudently restricted until more is known.

We conclude that endoscopic stenting and minor papilla sphincterotomy are technically feasible and may be clini-

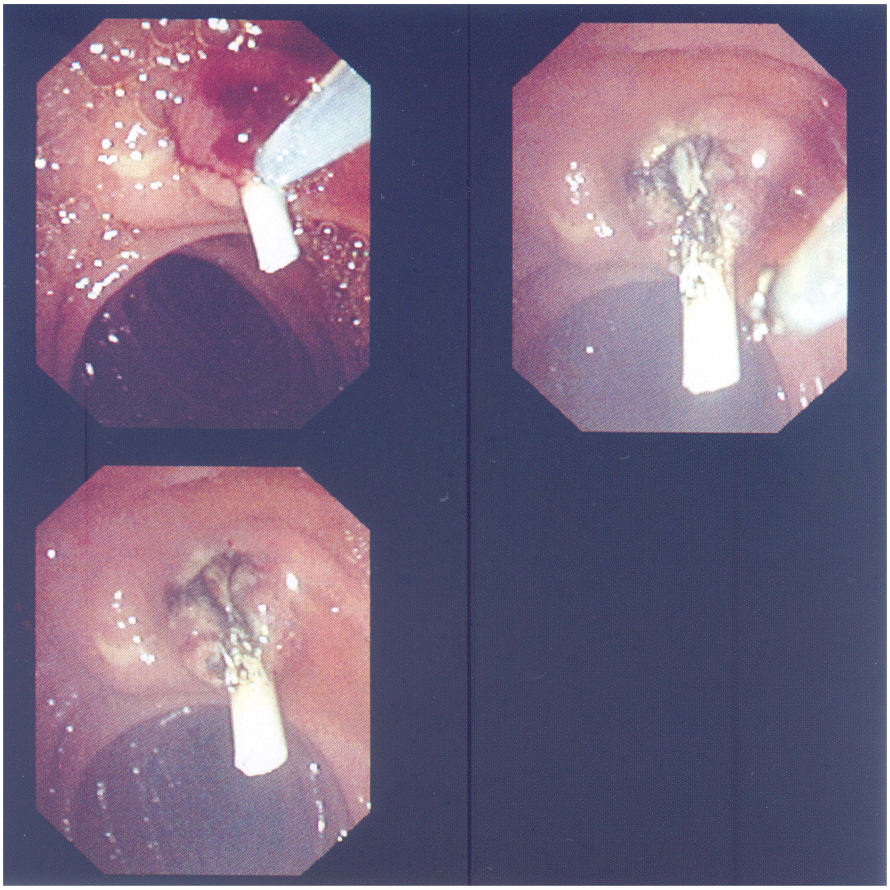

**Figure 6** Videoendoscopic sequence of minor papilla sphincterotomy performed with a stent in place. (*Upper panels* the needle knife cuts over the stent as a guide in the 11 o'clock orientation, (*lower panel*) after completion of the sphincterotomy.

cally effective in symptomatic patients with pancreas divisum. Endoscopic therapy of the pancreas, however, is limited by complications particularly related to stent migration and ductal changes. Investigators continue to refine patient selection, stent design, and duration of stenting. Endoscopic therapy of the pancreas should prudently be restricted to expert centers where clinical trials are being performed.

### REFERENCES

1. Millbourn E. Calibre and appearance of the pancreatic ducts and the relevant clinical problems: a roentgenographic and anatomical study: Acta Chir Scand 1959–1960;118:286–303
2. Cotton PB. Congenital anomaly of pancreas divisum as a cause of obstructive pain and pancreatitis. Gut 1980;21:105–14
3. Satterfield ST, McCarthy JH, Geenen JE, et al. Clinical experience in 82 patients with pancreas divisum: preliminary results of manometry and endoscopic therapy. Pancreas 1988;3:248–53
4. Delhaye M, Engelholm L, Cremer M. Pancreas divisum: congenital anatomic variant or anomaly? Gastroenterology 1985;89:951–958
5. Bernard JP, Sahel J, Giovanni M, et al. Pancreas divisum is a probable cause of acute pancreatitis: a report of 137 cases. Pancreas 1990;5:248–54
6. Cotton PB. Pancreas divisum: curiosity or culprit? Gastroenterology 1985;89:1431–1435
7. Carr-Locke DL. Pancreas divisum: the controversy goes on? Endoscopy 1991;23:88–90
8. Blair AJ, Russell CG, Cotton PB. Resection for pancreatitis in patients with pancreas divisum. Ann Surg 1984;200:590–594
9. Warshaw AL, Simeone JF, Schapiro RH, et al. Evaluation and treatment of the dominant dorsal duct syndrome. Am J Surg 1990;159:59–64
10. Keith RG, Shapero TF, Saibil FG, et al. Dorsal duct sphincterotomy is effective long-term treatment of acute pancreatitis with pancreas divisum. Surgery 1989;106:660–667
11. Siegel JH, Ben-Zvi JS, Pullano W, et al. Effectiveness of endoscopic drainage for pancreas divisum: endoscopic and surgical results in 31 patients. Endoscopy 1990;22:129–33.
12. Lans JI, Geenen JE, Johanson JF, et al. Endoscopic therapy in patients with pancreas divisum and acute pancreatitis: a prospective, randomized, controlled clinical trial. Gastrointest Endosc 1992;38:430–434
13. Lehman GA, Sherman S, Nisi R, et al. Pancreas divisum: results of minor papilla sphincterotomy. Gastrointest Endosc 1993;39:1–8
14. Siegel JH, Cooperman AM, Pullano W, et al. Pancreas divisum: observation, endoscopic drainage, and surgical treatment results in 65 patients. Surg Laparosc Endosc 1993;3:281–85.
15. Cotton PB, Lehman G, Vennes J, et al. Endoscopic sphincterotomy complications and their management: an attempt at consensus. Gastrointest Endosc 1991;31:383–393

16. Russel RCG, Wong NW, Cotton PB. Accessory sphincterotomy (endoscopic and surgical) in patients with pancreas divisum. Br J Surg 1984;71:954–957
17. McCarthy JH, Geenen JE, Hogan WJ. Endoscopic treatment in nonmalignant pancreatic disease. In Jacobson I. M., (ed): ERCP: diagnostic and therapeutic applications. New York: Elsevier, 1989:189–201
18. Soehendra N, Kempeneers I, Nam VC, et al. Endoscopic dilatation and papillotomy of the accessory papilla and internal drainage in pancreas divisum. Endoscopy 1986;18:129–132
19. Barkun AN, Jones S, Putnam WS, et al. Endoscopic treatment of patients with pancreas divisum and pancreatitis (abstr). Gastrointest Endosc 1990;36:206–207
20. Coleman SD, Cotton PB. Endoscopic accessory sphincterotomy and stenting in pancreas divisum (abstr). Gastrointest Endosc 1993;39:312
21. Sherman S, Hawes R, Nisi R, et al. Randomized controlled trial of minor papilla sphincterotomy in pancreas divisum patients with pain only (abstr). Gastrintest Endosc 1994;40:125
22. Sherman S, Lehman G, Nisi R, et al. Results for endoscopic sphincterotomy of the minor papilla for pancreas divisum (abstr). Gastrointest Endosc 1990;36:198
23. Johanson JF, Schmalz MJ, Geenen JE. Incidence and risk factors for biliary and pancreatic stent migration. Gastrointest Endosc 1992;38:341–346
24. Siegel JH, Veerappan A. Endoscopic mamagement of pancreatic disorders: potential risks of pancreatic prostheses. Endoscopy 1991;23:177–180
25. Siegel JH. Evaluation and treatment of acquired and congenital pancreatic disorders: endoscopic dilation and insertion of endoprostheses (abstr). Am J Gastroenterol 1983;78:696
26. Johanson JF, Schmalz MJ, Geenen JE. Simple modification of a pancreatic duct stent to prevent proximal migration. Gastrointest Endosc 1993;39:62–64
27. Kozarek RA. Pancreatic stents can induce ductal changes consistent with chronic pancreatitis. Gastrointest Endosc 1990;36:39–95
28. Burdick JS, Geenen JE, Venu RP, et al. Ductal morphological changes due to pancreatic stent therapy: a randomized controlled study (abstr). Am J Gastroenterol 1992;87:1281

